# ABCB1 and ABCG2 Overexpression Mediates Resistance to the Phosphatidylinositol 3-Kinase Inhibitor HS-173 in Cancer Cell Lines

**DOI:** 10.3390/cells12071056

**Published:** 2023-03-30

**Authors:** Chung-Pu Wu, Cheng-Yu Hung, Ya-Ju Hsieh, Megumi Murakami, Yang-Hui Huang, Tsung-Yao Su, Tai-Ho Hung, Jau-Song Yu, Yu-Shan Wu, Suresh V. Ambudkar

**Affiliations:** 1 Graduate Institute of Biomedical Sciences, College of Medicine, Chang Gung University, Taoyuan 33302, Taiwan; 2 Department of Physiology and Pharmacology, College of Medicine, Chang Gung University, Taoyuan 33302, Taiwan; 3 Molecular Medicine Research Center, College of Medicine, Chang Gung University, Taoyuan 33302, Taiwan; 4 Department of Obstetrics and Gynecology, Taipei Chang Gung Memorial Hospital, Taipei 10507, Taiwan; 5 Laboratory of Cell Biology, Center for Cancer Research, National Cancer Institute, NIH, Bethesda, MD 20892, USA; 6 Department of Medicine, College of Medicine, Chang Gung University, Taoyuan 33302, Taiwan; 7 Department of Obstetrics and Gynecology, Keelung Chang Gung Memorial Hospital, Keelung 20401, Taiwan; 8 Department of Biochemistry and Molecular Biology, College of Medicine, Chang Gung University, Taoyuan 33302, Taiwan; 9 Liver Research Center, Linkou Chang Gung Memorial Hospital, Taoyuan 33302, Taiwan; 10 Department of Chemistry, Tunghai University, Taichung 40704, Taiwan

**Keywords:** multidrug resistance, ABCB1, ABCG2, PI3K, HS-173

## Abstract

Constitutive activation of the phosphoinositide-3-kinase (PI3K)/Akt signaling pathway is crucial for tumor growth and progression. As such, this pathway has been an enticing target for drug discovery. Although HS-173 is a potent PI3K inhibitor that halts cancer cell proliferation via G2/M cell cycle arrest, the resistance mechanisms to HS-173 have not been investigated. In this study, we investigated the susceptibility of HS-173 to efflux mediated by the multidrug efflux transporters ABCB1 and ABCG2, which are two of the most well-known ATP-binding cassette (ABC) transporters associated with the development of cancer multidrug resistance (MDR). We found that the overexpression of ABCB1 or ABCG2 significantly reduced the efficacy of HS-173 in human cancer cells. Our data show that the intracellular accumulation of HS-173 was substantially reduced by ABCB1 and ABCG2, affecting G2/M arrest and apoptosis induced by HS-173. More importantly, the efficacy of HS-173 in multidrug-resistant cancer cells could be recovered by inhibiting the drug-efflux function of ABCB1 and ABCG2. Taken together, our study has demonstrated that HS-173 is a substrate for both ABCB1 and ABCG2, resulting in decreased intracellular concentration of this drug, which may have implications for its clinical use.

## 1. Introduction

The phosphoinositide-3-kinase (PI3K)/Akt signaling pathway has a critical role in regulating cell cycle progression, cell differentiation, and proliferation [1]. Constitutive activation of the PI3K/Akt pathway, through various mechanisms such as gene amplification or mutations of receptor tyrosine kinases, is common and crucial to the development of many types of cancer [2]. In addition, alterations of the PI3K/Akt signaling pathway are frequently associated with acquired cancer drug resistance [3,4]. Therefore, the PI3K/Akt pathway is considered one of the most desirable targets for cancer therapy [1]. Numerous pan-PI3K inhibitors and isoform-selective PI3K inhibitors, including the US Food and Drug Administration (FDA)-approved drugs idelalisib, alpelisib, and copanlisib, have been developed over the past decade [5,6]. These PI3K inhibitors are effective, both as single agents and in combination with cytotoxic anticancer drugs, for the treatment of solid tumors and blood cancers [5,6]. Consequently, many novel PI3K inhibitors are being developed, with some currently in clinical trials [7]. However, the emergence of drug resistance may limit their clinical use.

HS-173 is a novel imidazopyridine phosphatidylinositol 3-kinase alpha (PI3Kα) inhibitor [8]. Experimental data demonstrated that HS-173 could inhibit the proliferation of human non-small cell lung cancer (NSCLC) cells, human Hep3B hepatoma, and human SkBr3 breast cancer cells through the induction of G2/M phase cell cycle arrest and apoptosis [8,9]. Moreover, studies have reported that HS-173 could induce cell death in head and neck squamous cell carcinoma (HNSCC) cells [10], and the effect was enhanced by inhibitors of KDM4 and KDM6 histone lysine demethylases [11]. Through simultaneous targeting of Raf/MEK/ERK and PI3K/Akt signaling pathways, the synergistic effects of HS-173 with sorafenib against the proliferation of pancreatic cancer cells [12] and with trametinib against the proliferation of HNSCC cells [13] have been reported. More importantly, while examining the combination regimens of inhibitors of PI3K and the epidermal growth factor receptor (EGFR) for the treatment of HNSCC, the combination of HS-173 and afatinib, an irreversible EGFR/ERBB2 dual inhibitor, exhibited the highest synergistic effect in most models tested [14]. Unfortunately, several mechanisms that are known to contribute toward the development of resistance to PI3K inhibitors, such as the concurrent activation of survival-related pathways [15] and drug extrusion by efflux pumps [16,17], could potentially reduce the efficacy of HS-173 in cancer cells. 

The human ATP-binding cassette (ABC) drug transport proteins ABCB1 (P-glycoprotein/MDR1) [18] and ABCG2 (ABCP/BCRP/MXR) [19] are known to utilize the energy derived from ATP hydrolysis to translocate an extensive range of substrate drugs across biological membranes [20,21]. Consequently, the absorption, distribution, metabolism, and elimination (ADME) of a large number of chemotherapeutic drugs are significantly affected by ABCB1 and ABCG2, since they are highly expressed in liver hepatocytes and intestinal epithelial cells, as well as in cells forming important barrier sites such as the blood–brain barrier (BBB) and the blood–placenta barrier (BPB) [20,22,23]. More importantly, oftentimes ABCB1 and/or ABCG2 overexpression gives rise to the development of multidrug resistance (MDR) in cancer cells [21]. Clinical evidence has shown that high expression of ABCB1 and/or ABCG2 is linked with the MDR phenotype in advanced NSCLC [24], metastatic breast cancer [25], acute lymphocytic leukemia (ALL), acute myelogenous leukemia (AML) [26,27,28], chronic lymphocytic leukemia (CLL) [29], and multiple myeloma (MM) [30,31]. 

In addition to conventional cytotoxic anticancer drugs [20], ABCB1 and ABCG2 are known to reduce the efficacy of molecularly targeted drugs, including tyrosine kinase inhibitors (TKIs) [32,33], serine –threonine kinase inhibitors (STKs) [34,35,36,37], anaplastic lymphoma kinase (ALK) inhibitors [38], histone deacetylase (HDAC) inhibitors [39,40], and PI3K inhibitors [16,17,41]. Consequently, patients with high ABCB1- or ABCG2-expressing multidrug-resistant tumors are usually insensitive to chemotherapy, resulting in cancer relapse and treatment failure [21,42].

As the overexpression of ABCB1 and ABCG2 is known to be a common mechanism responsible for the development of drug resistance in cancer cells [21], the purpose of this study is to determine whether HS-173 is susceptible to efflux in human cancer cells induced by ABCB1 and ABCG2. We demonstrated that the drug transport activity of ABCB1 and ABCG2 could extensively lower the intracellular accumulation of HS-173 and consequently reduce its ability to induce G2/M phase cell cycle arrest and apoptosis in human cancer cells. Furthermore, the efficacy of HS-173 could be recovered through inhibiting the drug-efflux function of ABCB1 and ABCG2 in multidrug-resistant cancer cells. In summary, our data show the interactions of HS-173 with ABCB1 and ABCG2, as well as providing experimental evidence that the overexpression of ABCB1 or ABCG2 in cancer cells is a novel mechanism of acquired resistance to the PI3K inhibitor HS-173. Combination therapy using HS-173 with a modulator of ABCB1 or ABCG2 may be essential to improve the clinical efficacy of HS-173 and should be further examined in the future.

## 2. Materials and Methods

### 2.1. Chemicals and Cell Culture Conditions

The Dulbecco’s modified Eagle’s medium (DMEM), Iscove’s modified Dulbecco’s medium (IMDM), Rosewell Park Memorial Institute (RPMI) 1640 medium, Fetal calf serum (FCS), phosphate-buffered saline (PBS), _L_-glutamine, trypsin-EDTA, penicillin, and streptomycin were acquired from Gibco, Invitrogen (Carlsbad, CA, USA). The HS-173 was acquired from Selleckchem (Houston, TX, USA). The Tools Cell Counting Kit (CCK-8) was acquired from Biotools Co., Ltd. (Taipei, Taiwan). The Annexin V: Fluorescein isothiocyanate (FITC) Apoptosis Detection Kit was acquired from BD Pharmingen (San Diego, CA, USA). The Ko143, tariquidar, and other chemicals were purchased from Sigma-Aldrich (St. Louis, MO, USA) unless stated otherwise. The KB-3-1, KB-V1, MCF-7, MCF7-FLV1000, H460, H460-MX20, pcDNA3.1-HEK293, human ABCB1-transfected HEK293 (MDR19-HEK293) [43], and human ABCG2-transfected HEK293 (R482-HEK293) [44] were sustained in DMEM supplemented with 10% FCS, 2 mM _L_-glutamine, and 100 units of penicillin/streptomycin/mL. The OVCAR-8, NCI-ADR-RES, S1, and S1-MI-80 cells were sustained in RPMI-1640 supplemented with 10% FCS, 2 mM _L_-glutamine, and 100 units of penicillin–streptomycin/mL. The KB-V1 cells were sustained in medium with the addition of 1 µg/mL vinblastine [45]; the MCF7-FLV1000 cells were sustained in medium with the addition of 1 µg/mL flavopiridol [46]; the H460-MX20 cells were sustained in medium with the addition of 20 nM mitoxantrone [47]; the HEK293-transfected lines were sustained in medium with the addition of 2 mg/mL G418 [48]; the NCI-ADR-RES cells were sustained in medium with the addition of 0.85 μM doxorubicin [49]; and the S1-MI-80 cells were sustained in medium with addition of 80 μM mitoxantrone [50]. All cell lines were kept in 5% CO_2_ humidified air at 37 °C and placed in drug-free medium 7 days prior to assay.

### 2.2. Cell Viability Assays

The cytotoxicity of HS-173 and other chemotherapeutic agents was determined by MTT and CCK-8 assays. Briefly, the cells were plated into 96-well flat-bottom plates in culture medium at in 5% CO_2_ humidified air 37 °C for 24 h for the cells to attach. Following treatment with HS-173 or various drug combinations for an additional 72 h, the cells were subsequently developed using MTT [51] or CCK-8 reagent as described previously [48]. The IC_50_ values were calculated using the fitted concentration–response curves obtained from more than three independent experiments, and the extent of resistance was presented as a resistance factor (RF) value, determined by dividing the IC_50_ value of HS-173 in ABCB1- or ABCG2-expressing cells by the IC_50_ value of HS-173 in the respective parental cells. 

### 2.3. Assay to Determine Fluorescent Drug Transport

The accumulation of calcein, a fluorescent product of a known substrate of ABCB1 calcein-AM [52] in KB-3-1, KB-V1, OVCAR-8, NCI-ADR-RES, pcDNA3.1-HEK293, and MDR19-HEK293, or pheophorbide A (PhA), a known fluorescent substrate of ABCG2 [53] in S1, S1-MI-80, MCF-7, MCF7-FLV1000, pcDNA3.1-HEK293, and R482-HEK293, was determined as described previously [53,54]. Briefly, the cells were trypsinized and collected by centrifugation at 500× *g*, then resuspended in IMDM supplemented with 5% FCS. Calcein-AM or PhA was added to 3 × 10^5^ cells in 4 mL of IMDM for 10 min or 1 h, respectively, in the presence of DMSO (control), HS-173, and tariquidar or Ko143, reference inhibitors for ABCB1 and ABCG2. The relative fluorescence intensity was detected (485 nm excitation and 535 nm emission for calcein, and 395 nm excitation and 670 nm emission for PhA) and analyzed using a FACSort flow cytometer equipped with Cell Quest software version 3.3 (Becton-Dickinson Biosciences, San Jose, CA, USA) as described previously [35,53].

### 2.4. Cell Cycle Analysis

We used a standard propidium iodide (PI) staining method to determine the effect of HS-173 on cell cycle arrest in KB-3-1, KB-V1, S1, and S1-MI-80 cancer cell lines. Briefly, the cells were treated with DMSO or HS-173 with or without the addition of tariquidar or Ko143, reference inhibitors for ABCB1 and ABCG2, for 24 h and were later harvested in PBS and fixed in ethanol overnight. After being washed with PBS, the cells were treated with 0.5% TritonX-100 and 0.05% RNase in PBS at 37 °C for 1 h. The cells were washed and subsequently incubated in 50 μg/mL of PI at 4 °C for 20 min before analysis. A FACSort flow cytometer equipped with CellQuest software was used to analyze the cells as described previously [35,37,41].

### 2.5. Apoptosis Assay

To determine the apoptotic effect of HS-173 on KB-3-1, KB-V1, S1, and S1-MI-80 cancer cells, we used the conventional annexin V–FITC and PI staining method according to the instructions of the manufacturer (BD Pharmingen) and the method reported by Anderson et al. [55]. Briefly, the cells were treated with DMSO (control), tariquidar or Ko143 alone, HS-173 alone, or the combination of HS-173 and tariquidar or Ko143 as indicated for 48 h before we stained the cells with annexin V–FITC (1.25 µg/mL) and PI (0.1 mg/mL) at room temperature for 15 min. FACScan equipped with CellQuest software was used to analyze the labeled cells as described previously [56]. 

### 2.6. HS-173 Accumulation Assay and HPLC-MS/MS Analysis

The intracellular accumulation of HS-173 in OVCAR-8, NCI-ADR-RES, S1, and S1-MI-80 cancer cells was determined and quantified by the LC-MS/MS method as previously described [39] with slight modification. Briefly, 2 × 10^6^ cells were incubated with 10 μM HS-173 with or without the addition of 10 μM tariquidar or Ko143 for 1 h at 37 °C, washed twice with cold PBS, later harvested, and extracted with three volumes of methanol and stored overnight at −20 °C. Cell lysates with methanol extraction were centrifuged (12,000 rpm) for 30 min at 4 °C, and the supernatants were dried with a speed vacuum and reconstituted with 0.1% formic acid in 50% methanol. The samples were analyzed using a Waters UPLC (ultra-performance liquid chromatography) system on a BEH C18 column (130 Å, 1.7 µm, 1 × 100 mm, Waters Corp., Milford, MA, USA) coupled with HCT ultra (Bruker Daltonik GmbH, Bremen, Germany) by selected reaction monitoring (SRM) in positive mode. Mobile phase A consisted of water containing 0.1% formic acid, while mobile phase B consisted of acetonitrile containing 0.1% formic acid. The flow rate was 60 µL/min with a constant column temperature of 40 °C. The linear gradient method was: 0 min, 10% B; 2.5 min, 60% B; 4.5 min, 95% B, and holding 95% B for 4 min, and then finally, to 10% B and then equilibrated for 3 min. The optimal ion transition of HS-173 was set for the precursor ion *m*/*z* 423.2 with a 4 Da mass error, and the peak area of the specific fragment ion *m*/*z* 395.1 was integrated to quantify intracellular HS-173 accumulation using DataAnalysis 4.2 (Bruker Corporation). The calibration curve for HS-173 was prepared from HS-173 stock by serial dilution with equal amounts of cell lysate extract. Six different concentrations were generated, ranging from 0.05 to 5 pmol/μL.

### 2.7. ATPase Assays

Vanadate (Vi)-sensitive ABCB1-specific or ABCG2-specific ATPase activity was measured by endpoint P_i_ assay using membrane vesicles prepared from ABCB1-overexpressing or ABCG2-overexpressing High Five insect cells (Invitrogen, Carlsbad, CA, USA) as described previously [17,57].

### 2.8. Docking Analysis

The energy minimization of the chemical structure of HS-173, inward-open cryo-EM structures of ABCB1 (PDB:6QEX) [58] and ABCG2 (PDB:6VXH) [59], was conducted with the CHARMM force field at pH 7.4 using BIOVIA Discovery Studio 4.0 as previously described [60]. The docking of HS-173 with both ABCB1 and ABCG2 was performed by the CDOCKER module of the same software. 

### 2.9. Quantification and Statistical Analysis

The experimental data are shown as mean ± standard error of the mean (SEM), and the IC_50_ values were calculated as the mean ± standard deviation (SD) calculated from more than three independent experiments. The curves were plotted with GraphPad Prism software version 5.01 (La Jolla, CA, USA), and statistical analysis was performed using KaleidaGraph software version 4.5 (Reading, PA, USA). A two-tailed Student’s *t*-test was used to analyze the difference between the mean values of experiment and the control or the improvement in fit, labeled with asterisks as “statistically significant” if the probability, *p*, was less than 0.05.

## 3. Results

### 3.1. The Cytotoxicity of HS-173 Is Significantly Reduced in Cells Overexpressing ABCB1 or ABCG2

**Figure 1:**
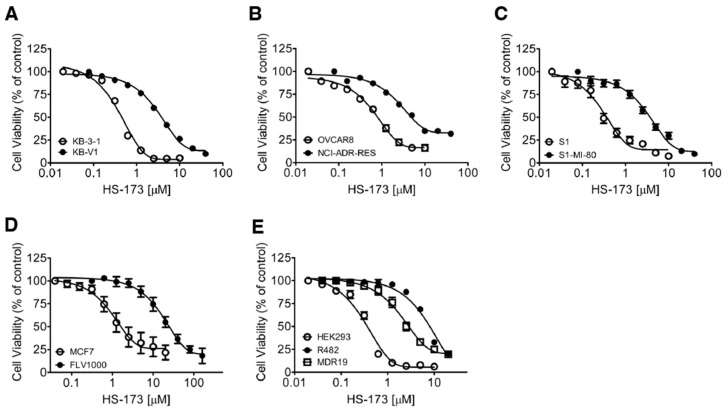
Dose–response curves for parental and multidrug-resistant cell lines overexpressing ABCB1 or ABCG2 treated with HS-173. The cytotoxicity of HS-173 in (**A**) the parental human KB-3-1 epidermal cancer cell line (open circles) and its ABCB1-overexpressing multidrug-resistant subline KB-V1 (filled circles); (**B**) parental human OVCAR-8 ovarian cancer cell line (open circles) and its ABCB1-overexpressing multidrug-resistant subline NCI-ADR-RES (filled circles); (**C**) parental human S1 colon cancer cell line (open circles) and its ABCG2-overexpressing multidrug-resistant subline S1-MI-80 (filled circles); (**D**) parental human MCF-7 breast cancer cell line (open circles) and its ABCG2-overexpressing multidrug-resistant subline MCF7-FLV1000 (filled circles); as well as (**E**) the parental HEK293 cells (open circles) and HEK293 cells transfected with human ABCB1 (MDR19-HEK293, open squares) or human ABCG2 (R482-HEK293, filled circles). Points, mean values from more than three independent experiments; bars, SEM.

The cytotoxicity of HS-173 in several pairs of ABCB1- and ABCG2-overexpressing cancer cell lines and the respective parental cell lines was established to elucidate the impact of ABCB1 and ABCG2 on the efficacy of HS-173 in cancer cells. The calculated IC_50_ values for HS-173 and the resistance factor (RF) values, representing the extent of resistance to HS-173 mediated by ABCB1 or ABCG2, are summarized in Table 1. First, we found from the data that the ABCB1-overexpressing KB-V1 human epidermal cancer cells were significantly resistant to HS-173 treatment (RF = 11) as compared to the parental KB-3-1 cancer cells (Figure 1A). In addition, the ABCB1-overexpressing NCI-ADR-RES human ovarian cancer cells were resistant to HS-173 (RF = 9) as compared to the parental OVCAR-8 cancer cells (Figure 1B). Similarly, the ABCG2-overexpressing S1-MI-80 human colon cancer cells, MCF7-FLV1000 human breast cancer cells, and H460-MX20 lung cancer cells were significantly resistant to HS-173 as compared to the parental S1 (Figure 1C), MCF7 (Figure 1D), and H460 cells, with calculated RF values of 10, 12, and 5, respectively. In addition, we determined the cytotoxicity of HS-173 in parental HEK293 cells, ABCB1-transfected MDR19-HEK293 cells, and ABCG2-transfected R482-HEK293 cells to confirm our findings. As shown in Figure 1E, both MDR19-HEK293 and R482-HEK293 cell lines were significantly resistant to HS-173 as compared to the parental HEK293 cell lines, with calculated RF values of 10 and 26, respectively. The results of the parental HEK293 cells being 10-fold and 26-fold more sensitive to HS-173 treatment as compared to ABCB1-transfected MDR19-HEK293 and ABCG2-transfected R482-HEK293 supported our finding that ABCB1 and ABCG2 confer resistance to HS-173. Next, we determine whether we could re-sensitize ABCB1-overexpressing KB-V1 and MDR19-HEK293 cells, as well ABCG2-overexpressing S1-MI-80 and R482-HEK293 cells to HS-173 with tariquidar or Ko143, reference inhibitors for ABCB1 and ABCG2, respectively. As shown in Table 2, the chemosensitivity of ABCB1- and ABCG2-overexpressing multidrug-resistant cells to HS-173 was significantly restored by both tariquidar and Ko143. 

### 3.2. Effect of ABCB1 and ABCG2 Overexpression on HS-173-Induced G2/M Cell Cycle Arrest and Apoptosis in Human Cancer Cells

**Figure 2:**
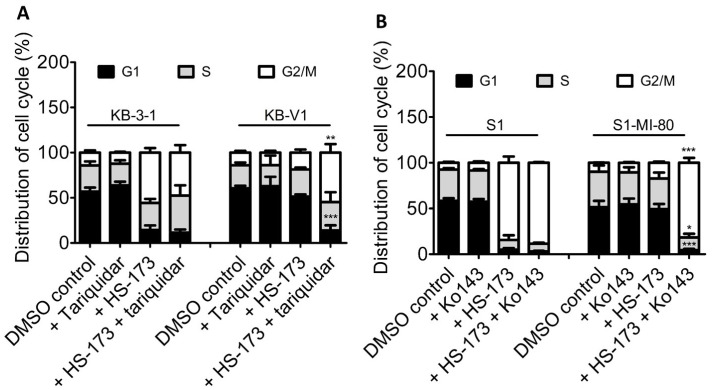
Differential effect of HS-173 on G2/M cell cycle arrest in drug-sensitive and multidrug-resistant cancer cells overexpressing ABCB1 or ABCG2. (**A**) KB-3-1 and KB-V1 cells were treated with DMSO (control), 1 μM tariquidar, 2 μM HS-173, or a combination of HS-173 and tariquidar; (**B**) S1 and S1-MI-80 cells were treated with DMSO (control), 1 μM Ko143, 2 μM HS-173, or a combination of HS-173 and Ko143 for 24 h before harvesting for cell cycle analysis, as described in the Materials and Methods section. * *p* < 0.05; ** *p* < 0.01; *** *p* < 0.001.

Knowing that ABCB1 and ABCG2 could reduce the cytotoxicity of HS-173 in cancer cell lines, we next examined the impact of ABCB1 and ABCG2 on G2/M cell cycle arrest and apoptosis induced by HS-173, which are characteristic effects of HS-173 treatment [8,9] in human cancer cell lines. First, we compared the effect of 2 μM HS-173 on cell cycle phase distribution in KB-3-1, KB-V1, S1, and S1-MI-80 cancer cell lines with or without the addition of a reference inhibitor of ABCB1 or ABCG2. As expected, HS-173 substantially induced G2/M cell cycle arrest in KB-3-1 cells, from 14.1 ± 2.5% basal to 55.7 ± 5.1% (Figure 2A), and in S1 cells, from 7.4 ± 1.1% basal to 84.4 ± 6.8% (Figure 2B). In contrast, G2/M arrest induced by HS-173 was considerably less in ABCB1-overexpressing KB-V1 cells, from 14.0 ± 2.0% basal to 18.6 ± 3.4% (Figure 2A), and in ABCG2-overexpressing S1-MI-80 cells, from 9.8 ± 0.3% basal to 17.2 ± 1.3% (Figure 2B). More importantly, tariquidar and Ko143 could restore HS-173-induced G2/M cell cycle arrest induced in KB-V1 cells to 54.7 ± 9.4% (Figure 2A) and S1-MI-80 cells to 81.7 ± 5.2% (Figure 2B). 

**Figure 3:**
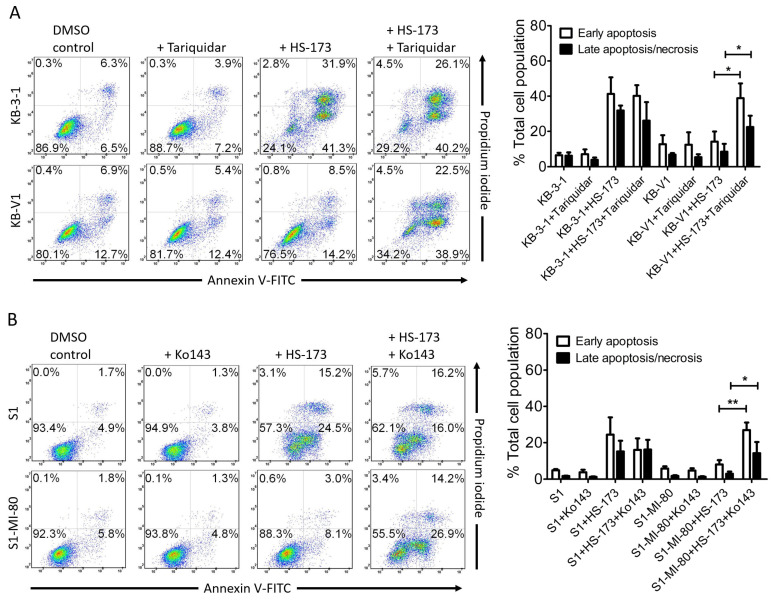
Differential effect of HS-173-induced apoptosis in drug-sensitive cancer cells and multidrug-resistant cancer cells overexpressing ABCB1 or ABCG2. (**A**) KB-3-1 and KB-V1 cells were treated with DMSO (control), 1 μM tariquidar, 2 μM HS-173, or a combination of HS-173 and tariquidar; (**B**) S1 and S1-MI-80 cells were treated with DMSO (control), 1 μM Ko143, 2 μM HS-173, or a combination of HS-173 and Ko143 for 48 h, as described in the Materials and Methods section. The cells were later processed using the Annexin V-FITC and PI staining method and analyzed by means of flow cytometry. Representative dot plots (left panel) and quantified values (right panel) are mean values ± SD calculated from more than three independent experiments. * *p* < 0.05; ** *p* < 0.01, versus the same treatment with the addition of tariquidar or Ko143.

Next, we found that HS-173 induced significant apoptosis in KB-3-1 cancer cells, from 13% basal to approximately 73% (Figure 3A), and in S1 cancer cells, from 7% basal to approximately 40% (Figure 3B). In contrast, HS-173 merely increased the level of apoptosis from 20% basal to approximately 23% in KB-V1 cancer cells (Figure 3A), and from 8% basal to approximately 11% in S1-MI-80 cancer cells (Figure 3B). Treatment with tariquidar or Ko143 was able to restore the activity of HS-173 to induce cell apoptosis in KB-V1 and S1-MI-80 cancer cells by inhibiting the drug transport function of ABCB1 and ABCG2, respectively. Notably, tariquidar or Ko143 alone did not induce significant apoptosis or G2/M cell-cycle arrest in any of the cell lines examined. Taken together, our results demonstrate that the efficacy of HS-173 was reduced by the drug-transport function of ABCB1 and ABCG2 in cancer cells.

### 3.3. Effect of ABCB1 and ABCG2 Overexpression on the Intracellular Accumulation of HS-173 in Cancer Cells

**Figure 4:**
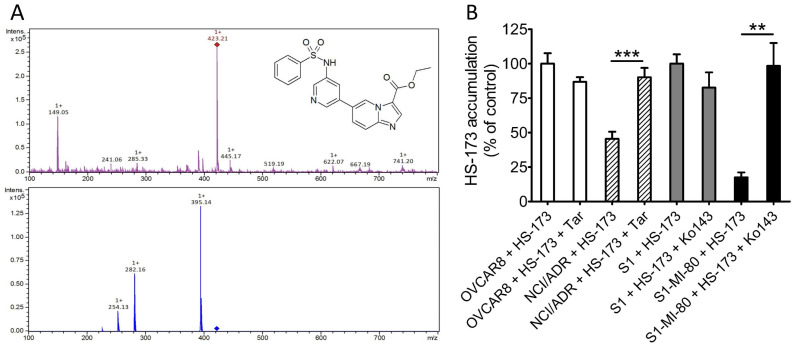
ABCB1- and ABCG2-mediated transport reduces the intracellular accumulation of HS-173 in human cancer cells. (**A**) The chemical structure and product ion mass spectra of HS-173 (precursor ion *m*/*z* 423.2 in positive mode). The fragment ion *m*/*z* 395.1 was selected for quantitative analysis. (**B**) Quantification of intracellular concentration of HS-173 by HPLC-MS/MS analysis in drug-sensitive OVCAR-8 cells (open bars), ABCB1-overexpressing NCI-ADR-RES (striped bars), drug-sensitive S1 (gray bars), and ABCG2-overexpressing S1-MI-80 (black bars) cells with or without the addition of 10 μM tariquidar or Ko143 as described in the Materials and Methods section. Values are mean values ± SD calculated from more than three independent experiments. ** *p* < 0.01 and *** *p* < 0.001 versus the treatment with tariquidar or Ko143.

As one of the most likely reasons for the decreased efficacy of HS-173 is the reduced intracellular concentration of HS-173 caused by ABCB1- and ABCG2-mediated drug efflux, the intracellular accumulation of HS-173 was determined in OVCAR-8, NCI-ADR-RES, S1, and S1-MI-80 cancer cells using the LC-MS/MS method (Figure 4A) as described previously [39]. As shown in Figure 4B, the intracellular concentration of HS-173 was considerably lower in ABCB1-overexpressing NCI-ADR-RES and ABCG2-overexpressing S1-MI-80 cancer cells as compared to the parental OVCAR-8 and S1 cancer cells. More importantly, tariquidar and Ko143 significantly restored the level of HS-173 in NCI-ADR-RES (from 45% to 90% control value) and S1-MI-80 cancer cells (from 18% to 98% control value), respectively. Our results indicate that HS-173 efflux mediated by ABCB1 and ABCG2 significantly contributed to the reduced efficacy of HS-173 in these cancer cells. 

### 3.4. HS-173 Attenuates Drug Transport Mediated by ABCB1 and ABCG2

**Figure 5:**
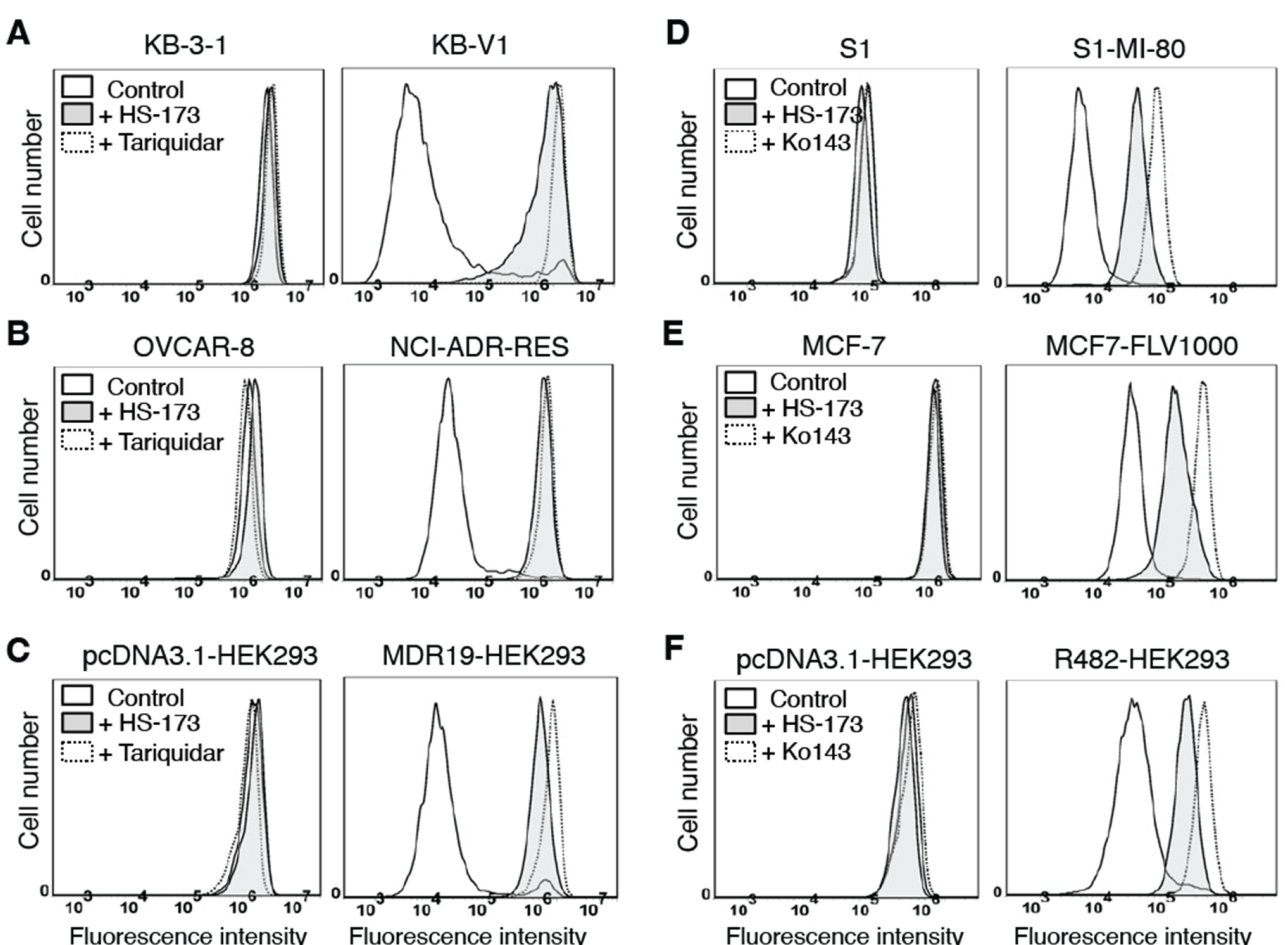
HS-173 inhibits ABCB1- and ABCG2-mediated drug transport. The accumulation of fluorescent calcein in the KB-3-1 cell line (**A**, left panel) and its ABCB1-overexpressing KB-V1 line (**A**, right panel); OVCAR-8 cell line (**B**, left panel) and its ABCB1-overexpressing NCI-ADR-RES (**B**, right panel) cells; HEK293 cells stably transfected with the pcDNA3.1 empty vector (**C**, left panel) and HEK293 cells stably transfected with human ABCB1 (**C**, right panel), or fluorescent pheophorbide A (PhA) in the S1 cell line (**D**, left panel) and its ABCG2-overexpressing S1-MI-80 line (**D**, right panel); MCF-7 cell line (**E**, left panel) and its ABCG2-overexpressing MCF7-FLV1000 line (**E**, right panel); HEK293 cells stably transfected with the pcDNA3.1 empty vector (**F**, left panel) and HEK293 cells stably transfected with human ABCG2 (**F**, right panel), measured without (solid lines) or with the addition of 40 μM HS-173 (shaded, solid lines) or 1 μM tariquidar or Ko143 (dotted lines) and analyzed immediately by means of flow cytometry, as described in the Materials and Methods section. Representative histograms of three independent experiments are shown.

The transport of one substrate mediated by ABCB1 or ABCG2 is often affected by the transport of another substrate [21,61,62]. Therefore, we studied the effect of HS-173 on ABCB1-mediated efflux of a known ABCB1 substrate calcein-AM [52] and the ABCG2-mediated efflux of a known ABCG2 substrate PhA [53] in cells overexpressing ABCB1 or ABCG2, respectively. We found that 40 μM HS-173 increased the fluorescence level of calcein, the fluorescent product of calcein-AM, in ABCB1-overexpressing KB-V1 (Figure 5A), NCI-ADR-RES (Figure 5B) and MDR19-HEK293 (Figure 5C) cells. Similarly, the fluorescence level of PhA in ABCG2-overexpressing S1-MI-80 (Figure 5D), MCF7-FLV1000 (Figure 5E), and R482-HEK293 (Figure 5F) cells was increased by 40 μM HS-173. Of note, neither HS-173 nor reference inhibitors of ABCB1 and ABCG2 had a substantial effect on the accumulation of calcein-AM and PhA in the respective parental cells (Figure 5A–F, left panels). Our results suggest that when HS-173 was being transported by ABCB1 and ABCG2, the transport of other drug substrates of ABCB1 and ABCG2 was inhibited.

Numerous studies have reported that MDR mediated by ABCB1 and/or ABCG2 could be reversed by certain drug substrates of ABCB1 and/or ABCG2 [62]. To this end, we examined the effect of HS-173 on MDR mediated by ABCB1 and ABCG2 in cancer cells overexpressing ABCB1 or ABCG2. As shown in Table 3, HS-173 at the maximum sub-toxic concentration of 100 nM had no significant effect on ABCB1-mediated resistance to well-established anticancer drug substrates of ABCB1 (colchicine, vincristine, or paclitaxel) [63] in ABCB1-overexpressing KB-V1 cancer cells. Similarly, HS-173 at the same concentration did not re-sensitize ABCG2-overexpressing S1-MI-80 cancer cells to the drug substrates of ABCG2 (mitoxantrone, topotecan or SN-38) [64,65,66]. In contrast, tariquidar and Ko143 completely reversed MDR mediated by ABCB1 and ABCG2 in these cancer cell lines. 

### 3.5. HS-173 Stimulates the ATPase Activity of ABCB1 and ABCG2

**Figure 6:**
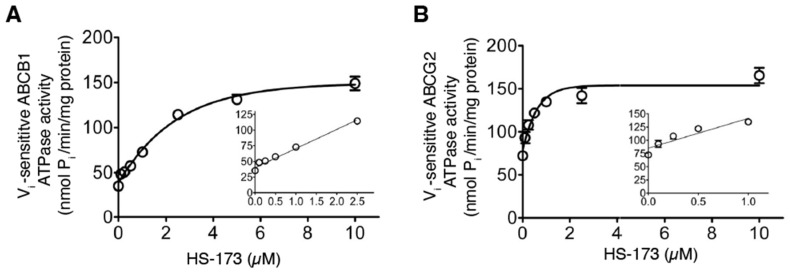
HS-173 stimulates the ATPase activity of ABCB1 and ABCG2. The effect of HS-173 (0–10 µM) on the vanadate (Vi)-sensitive ATP hydrolysis of (**A**) ABCB1 and (**B**) ABCG2, determined by endpoint Pi assay, as described previously [37,57]. Points, mean from a minimum of three independent experiments; bars, SEM.

Drug transport mediated by ABCB1 and ABCG2 is known to be coupled to the stimulation of the ATPase activity of ABCB1 and ABCG2 [67]. As such, we investigated the effect of HS-173 on the V_i_-sensitive ATPase activity of ABCB1 and ABCG2 as described in the Materials and Methods section. As shown in Figure 6A, HS-173 stimulated the ATPase activity of ABCB1 in a concentration-dependent manner, with a maximum stimulation of 423% of the basal value of 35.2 ± 2.8 nmoles P_i_/min/mg protein and a concentration to produce half of the maximum stimulation of approximately 1.7 μM. Similarly, the ABCG2-mediated ATP hydrolysis was stimulated by HS-173 in a concentration-dependent manner, with a maximum stimulation of 229% of the basal value of 72.3 ± 1.6 nmoles P_i_/min/mg protein and a concentration to produce half of the highest stimulation of approximately 0.4 μM (Figure 6B). Our results indicate that HS-173 is actively transported by both ABCB1 and ABCG2, which is consistent with the drug accumulation and cytotoxicity data.

### 3.6. Docking of HS-173 in the Drug-Binding Pocket of ABCB1 and ABCG2

**Figure 7:**
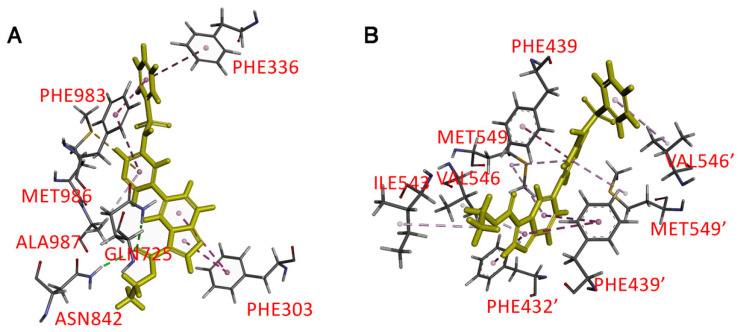
The lowest energy poses for the docking binding of HS-173 within the drug-binding pocket of (**A**) ABCB1 (PDB:6QEX) and (**B**) ABCG2 (PDB: 6VXH) were predicted using BIOVIA Discovery Studio 4.0 software as described in the Materials and Methods section. The molecular model of HS-173 is presented in (yellow) stick representation, and the atoms for interacting amino acid residues are colored: carbon (gray), oxygen (red), nitrogen (blue), hydrogen (light gray), and sulfur (yellow). Dashed lines represent the proposed interactions. The residues from monomer 2 of ABG2 are indicated with prime symbols.

To gain further insight into the interaction of HS-173 with the drug-binding pockets of ABCB1 and ABCG2, an in silico molecular docking analysis of HS-173 in the inward-open conformations of human ABCB1 (PDB:6QEX) [58] and ABCG2 (PDB: 6VXH) [59] was performed. The predicted hydrophobic interactions between HS-173 and several aromatic and hydrophobic residues located within the transmembrane domains of ABCB1 (Figure 7A) and ABCG2 (Figure 7B) were identified by analyzing poses with the lowest docking energy. Hydrophobic interactions were predicted in the substrate binding pocket of ABCB1 where Phe^303^ interacts with the dihydroimidazo [1,2-a]pyridine moiety, Phe^336^ interacts with the phenyl ring, and Phe^983^, Met^986^, and Ala^987^ interact with the pyridyl group of HS-173. There are also two possible hydrogen bonds between Gln^725^ and Asn^842^ with the ester carbonyl group on HS-173. The binding of HS-173 with ABCG2 was also predicted in the substrate/inhibitor binding pocket. Hydrophobic interactions were found between three aromatic moieties of HS-173 and Phe^432^, Phe^439^, Ile^542^, Val^546^, and Met^549^ on both monomers of the ABCG2 protein.

## 4. Discussion

The promising preclinical antiproliferative activity of HS-173 as a PI3K inhibitor, alone and together with other agents, has been demonstrated in a wide variety of cancers [8,9,10,11,12,13,14,68]. In addition to being a reference inhibitor of PI3K [8,14,69], additional pharmacological activities of HS-173 have been reported. HS-173 exhibited an anti-fibrotic activity in Peyronie’s disease (PD)-derived primary fibroblasts [70] and hepatic stellate cells (HSCs) [71]. Rumman et al. demonstrated that HS-173 suppresses the transforming growth factor (TGF)-β-induced epithelial–mesenchymal transition (EMT) and metastasis of human pancreatic cancer cells [72]. Park et al. found that HS-173 improves radiotherapy by attenuating DNA damage response mediated by the ataxia-telangiectasia mutated (ATM) and DNA-dependent protein kinase catalytic subunit (DNA-PKcs) in pancreatic cancer cells [73]. Kim et al. reported that HS-173 improves the delivery and efficacy of chemotherapeutic agents and reduces the number of metastatic lung nodules by improving the vascular structure and function of tumor vessels [74]. More recently, Park et al. identified HS-173 as a novel inducer of receptor-interacting kinase 3 (RIP3)-dependent necroptosis in lung cancer cells [68]. However, despite the many activities of HS-173 that have been reported, the possible mechanism of resistance to HS-173 in cancer cells, which could become a therapeutic challenge to clinicians, has not been explored.

As the first step in this study, we determined the cytotoxicity of HS-173 in multiple paired parental cancer cell lines and respective sublines expressing either ABCB1 or ABCG2. We found that regardless of the tissue of origin, the cytotoxicity of HS-173 was significantly reduced in multidrug-resistant sublines overexpressing ABCB1 or ABCG2 as compared to the respective parental lines. Similarly, HS-173 was less cytotoxic to HEK293 cells with ectopic expression of human ABCB1 or human ABCG2 as compared to the parental HEK293 cells (Table 1). Furthermore, we found that the effect of HS-173 on G2/M cell-cycle arrest and apoptosis was substantially reduced in ABCB1-overexpressing KB-V1 and ABCG2-overexpressing S1-MI-80 cancer cell lines when compared to what was observed with their respective parental cell lines (Figure 2 and Figure 3). More importantly, our results show that tariquidar and Ko143 can recover the sensitivity of ABCB1- and ABCG2-overexpressing cells to HS-173, supporting the notion that HS-173 resistance is caused by the drug efflux function of ABCB1 and ABCG2 (Table 2). As one of the most prevalent reasons for the lack of drug efficacy in multidrug-resistant cancer cells is due to reduced intracellular drug accumulation caused by active drug efflux mediated by ABCB1 or ABCG2 [21], the intracellular accumulation of HS-173 in parental cancer cell lines and respective sublines overexpressing ABCB1 or ABCG2 was determined using the LC-MS/MS method established previously [39]. As expected, we found a significantly reduced level of HS-173 in ABCB1-overexpressing NCI-ADR-RES and ABCG2-overexpressing S1-MI-80 sublines as compared to the drug-sensitive parental lines, which could be recovered by inhibiting the function of ABCB1 and ABCG2 (Figure 4).

Several studies have reported that some PI3K inhibitors are able to reverse MDR mediated by ABCB1 and/or ABCG2 in cancer cell lines [41,75,76]. For instance, Muthiah et al. demonstrated that the PI3K inhibitor ZSTK474 could potentiate the cytotoxicity of vinblastine and mitoxantrone in cancer cells overexpressing ABCB1 or ABCG2 by inhibiting the drug efflux function of both transporters [76]. Similarly, dactolisib reversed drug resistance mediated by ABCB1 and ABCG2 in AML [77] and mesothelioma cell lines [78], respectively. Others such as perifosine [79], BEZ235 [80], and DHW-211 [81] have been reported to reverse drug resistance in ABCB1-overexpressing breast cancer, ovarian cancer, and NSCLC cell lines, respectively. We found that although HS-173 could inhibit ABCB1-mediated efflux of calcein-AM and ABCG2-mediated PhA efflux at higher concentrations (Figure 5), HS-173 was unable to reverse the drug resistance mediated by ABCB1 or ABCG2 at sub-toxic concentrations (Table 3). The in silico molecular docking of HS-173 to the inward-open conformation of human ABCB1 (PDBID:6QEX) [58] and ABCG2 (PDBID:5NJ3) [82], which is the conformation of these proteins when a substrate binds to the substrate-binding pocket, supports the notion that HS-173 competes with the binding of other substrates of human ABCB1 and ABCG2 (Figure 7). Similar to other substrates, HS-173 stimulated the V_i_-sensitive ATPase activity of ABCB1 and ABCG2 (Figure 6) [17,34,35,39]. Our findings demonstrate that HS-173 is a transport substrate of both ABCB1 and ABCG2.

## 5. Conclusions

In conclusion, we suggest that there might be clinical implications for patients receiving HS-173 in treatment caused by acquired resistance to HS-173 mediated by ABCB1 and ABCG2 and that relevant areas of research should be further explored. Moreover, since both ABCB1 and ABCG2 have a significant impact on the ADME of a number of drugs [21,83], the potential effect of ABCB1 and ABCG2 on the oral bioavailability and distribution of HS-173 remains to be determined.

## Figures and Tables

**Table 1:** Cytotoxicity of HS-173 in drug-sensitive cells and multidrug-resistant cells overexpressing either human ABCB1 or ABCG2.

Cell Line	Type	Transporter Expressed	IC_50_ (μM) ^†^	RF ^‡^
KB-3-1	Epidermal	-	0.34 ± 0.08	1
KB-V1	Epidermal	ABCB1	3.69 ± 0.14 ***	11
OVCAR-8	Ovarian	-	0.88 ± 0.11	1
NCI-ADR-RES	Ovarian	ABCB1	7.64 ± 1.76 **	9
S1	Colon	-	0.36 ± 0.06	1
S1-MI-80	Colon	ABCG2	3.73 ± 0.33 ***	10
MCF7	Breast	-	2.03 ± 0.39	1
MCF7-FLV1000	Breast	ABCG2	24.09 ± 1.61 ***	12
H460	Lung	-	1.38 ± 0.37	1
H460-MX20	Lung	ABCG2	6.28 ± 1.18 **	5
pcDNA3.1-HEK293	-	-	0.28 ± 0.07	1
MDR19-HEK293	-	ABCB1	2.87 ± 0.31 ***	10
R482-HEK293	-	ABCG2	7.38 ± 1.37 ***	26

Abbreviations: RF, resistance factor. ^†^ IC_50_ values are mean ± SD calculated from dose–response curves obtained from more than three independent experiments using cytotoxicity assay as described in the Materials and Methods section. ^‡^ RF values were calculated by dividing IC_50_ values of HS-173 in multidrug-resistant cells by IC_50_ values of HS-173 in the respective parental cells. ** *p* < 0.01; *** *p* < 0.001.

**Table 2:** Effect of reference inhibitors of ABCB1 and ABCG2 on the cytotoxicity of HS-173 in cells overexpressing ABCB1 or ABCG2.

Cell Line	Mean IC_50_ ± SD [μM] ^†^		
	HS-173	HS-173 + Tariquidar	HS-173 + Ko143
KB-3-1	0.34 ± 0.08	0.31 ± 0.07	N.D.
KB-V1	3.69 ± 0.14	0.39 ± 0.07 ***	N.D.
S1	0.36 ± 0.06	N.D.	0.38 ± 0.06
S1-MI-80	3.73 ± 0.33	N.D.	0.70 ± 0.22 ***
pcDNA3.1-HEK293	0.28 ± 0.07	0.28 ± 0.08	0.24 ± 0.05
MDR19-HEK293	2.87 ± 0.31	0.23 ± 0.05 ***	N.D.
R482-HEK293	7.38 ± 1.37	N.D.	0.56 ± 0.13 **

Abbreviation: N.D., not determined. ^†^ IC_50_ values are mean ± SD calculated from dose–response curves obtained from more than three independent experiments, with or without the addition of 1 μM of tariquidar or Ko143. ** *p* < 0.01; *** *p* < 0.001.

**Table 3:** Effect of HS-173 on ABCB1- and ABCG2-mediated multidrug resistance in ABCB1- and ABCG2-overexpressing cancer cells.

Treatment	Concentration (nM)	Mean IC_50_ ± SD ^†^	
		**KB-3-1 (nM)**	**KB-V1 (μM)**
Colchicine	-	9.21 ± 3.15	1.81 ± 0.21
+ HS-173	50	8.02 ± 2.59	1.93 ± 0.14
+ HS-173	100	8.14 ± 2.73	1.80 ± 0.10
+ Tariquidar	1000	9.34 ± 3.27	11.38 ± 3.68 *** (nM)
Vincristine	-	3.15 ± 0.76	1.71 ± 0.33
+ HS-173	50	3.20 ± 0.65	1.91 ± 0.41
+ HS-173	100	2.78 ± 0.49	1.84 ± 0.33
+ Tariquidar	1000	2.11 ± 0.52	3.47 ± 0.75 *** (nM)
Paclitaxel	-	2.76 ± 0.86	4.78 ± 0.91
+ HS-173	50	2.16 ± 0.54	4.39 ± 0.51
+ HS-173	100	2.21 ± 0.58	3.98 ± 0.49
+ Tariquidar	1000	2.78 ± 0.87	3.44 ± 0.82 *** (nM)
		**S1 (nM)**	**S1-MI-80 (μM)**
Mitoxantrone	-	8.13 ± 1.63	64.43 ± 4.72
+ HS-173	50	6.11 ± 1.37	62.64 ± 7.01
+ HS-173	100	5.72 ± 1.26	61.46 ± 7.26
+ Ko143	1000	6.53 ± 1.41	1.12 ± 0.17 ***
Topotecan	-	71.63 ± 11.09	10.62 ± 2.77
+ HS-173	50	77.30 ± 11.95	9.70 ± 2.78
+ HS-173	100	78.00 ± 12.22	10.90 ± 3.13
+ Ko143	1000	75.81 ± 10.15	0.70 ± 0.15 **
SN-38	-	5.60 ± 0.81	3.24 ± 0.93
+ HS-173	50	6.70 ± 1.12	3.46 ± 0.92
+ HS-173	100	7.72 ± 1.31	3.38 ± 0.88
+ Ko143	1000	5.73 ± 0.88	47.59 ± 13.95 ** (nM)

Abbreviations: ^†^ IC_50_ values are mean ± SD calculated from dose–response curves obtained from three independent experiments using cytotoxicity assay as described in the Materials and Methods section. ** *p* < 0.01; *** *p* < 0.001.

## Data Availability

All data generated by this study are contained within the article.

## Abbreviations

ABC, ATP-binding cassette; PI3K, phosphatidylinositol 3-kinase; FCS, fetal calf serum; CCK-8, Cell Counting Kit-8; MTT, 3-(4,5-dimethylthiazol-yl)-2,5-diphenyllapatinibrazolium bromide; MDR, multidrug resistance; Vi, sodium orthovanadate; IMDM, Iscove’s modified Dulbecco’s medium; FR, fold reversal; RF, resistance factor.
